# T cells mediate autoantibody-induced cutaneous inflammation and blistering in epidermolysis bullosa acquisita

**DOI:** 10.1038/srep38357

**Published:** 2016-12-05

**Authors:** Katja Bieber, Mareike Witte, Shijie Sun, Jennifer E. Hundt, Kathrin Kalies, Sören Dräger, Anika Kasprick, Trix Twelkmeyer, Rudolf A. Manz, Peter König, Jörg Köhl, Detlef Zillikens, Ralf J. Ludwig

**Affiliations:** 1 Lübeck Institute of Experimental Dermatology (LIED), University of Lübeck, Ratzeburger Allee 160, D-23538 Lübeck, Germany; 2 Department of Immunology, Dalian Medical University, No9 West Section Lvshun S Rd, Liaoning Province, China; 3 Institute of Anatomy, University of Lübeck, Ratzeburger Allee 160, D-23538 Lübeck, Germany; 4 Department of Dermatology, Johannes Gutenberg-University Mainz, Saarstraße 21, D-55122 Mainz, Germany; 5 ISEF, University of Lübeck, Ratzeburger Allee 160, D-23538 Lübeck, Germany; 6 Department of Dermatology, University of Lübeck, Ratzeburger Allee 160, D-23538 Lübeck, Germany

## Abstract

T cells are key players in autoimmune diseases by supporting the production of autoantibodies. However, their contribution to the effector phase of antibody-mediated autoimmune dermatoses, i.e., tissue injury and inflammation of the skin, has not been investigated. In this paper, we demonstrate that T cells amplify the development of autoantibody-induced tissue injury in a prototypical, organ-specific autoimmune disease, namely epidermolysis bullosa acquisita (EBA) – characterized and caused by autoantibodies targeting type VII collagen. Specifically, we show that immune complex (IC)-induced inflammation depends on the presence of T cells – a process facilitated by T cell receptor (TCR)γδ and NKT cells. Because tissue damage in IC-induced inflammation is neutrophil-dependent, we further analyze the interplay between T cells and neutrophils in an experimental model of EBA. We demonstrate that T cells not only enhance neutrophil recruitment into the site of inflammation but also interact with neutrophils in lymphatic organs. Collectively, this study shows that T cells amplify the effector phase of antibody-induced tissue inflammation.

T cells are essential regulators of host defense and exhibit direct cytotoxic as well as regulatory properties. The presence or absence of pro-inflammatory compared with regulatory T cell subsets affects the development and outcome of inflammatory reactions. Misbalance of T cell populations leads to autoimmune disorders, including systemic lupus erythematosus (SLE), different autoimmune bullous dermatoses (AIBDs) and rheumatoid arthritis (RA)^1^,^2^,^3^. In these diseases, the contribution of T cells to antibody production and maintenance of the autoimmune response has clearly been demonstrated^4^,^5^.

In recent decades, the understanding of autoantibody-induced tissue injury has greatly improved. However, the role of T cells during the effector phase of autoimmune skin blistering diseases, i.e., tissue injury and inflammation in the targeted organs, is not completely understood. In this study, we investigated the role of T cells during this phase, using a mouse model of epidermolysis bullosa acquisita (EBA), a prototypical organ-specific autoimmune disease^6^,^7^. EBA is caused by autoantibodies directed against type VII collagen (COL7), an integral component of anchoring fibrils^8^. Animal models, employing antibody transfer into mice^9^,^10^, have added to a greater understanding of the mechanisms leading to blistering in EBA^9^,^11^,^12^. Based on the current understanding of EBA pathogenesis, the effector phase of EBA is predominantly driven by neutrophils – their depletion leading to a complete absence of experimental EBA^13^. With regard to T cell involvement during this phase, *in vivo* and *in vitro* data have been contradictory. *In vivo* data indicated a T cell-independent process: Transfer of total IgG isolated from rabbits that had been immunized with COL7 into T cell-deficient mice induced subepidermal blistering^9^. However, in that study, no wild-type control for evaluation of the extent of blistering was included. In other antibody transfer models, inflammation was more severe when T cells were absent. Specifically, in collagen type II (CII)-antibody transfer-induced murine arthritis, T cell receptor (TCR)β-deficient mice developed a higher arthritis incidence and severity score than wild-type animals^14^. In RA models such as the K/BxN model of arthritis and autoimmune cardiomyopathy^15^, the absence of functional T cells had no effect on disease development^16^,^17^.

By contrast to the above-presented *in vivo* data, *in vitro* experiments support a crosstalk between neutrophils and T cells, promoting inflammatory responses^12^. For example, neutrophils attract Th1 and Th17 cells *in vitro* by releasing chemokines^18^. Further *in vitro* data demonstrate the influence of activated T cells on the survival and recruitment of neutrophils. Indeed, activated CD4 + and CD8 + T cells, including Th17 cells, produce cytokines (IFNγ, GM-CSF and TNF) that modulate neutrophil survival and the expression of activation markers in culture systems^19^. Similarly, γδT cells strongly promote neutrophil survival and activation, as determined by the upregulation of CD64, HLA-DR, TNF and IL-17 production^20^,^21^. Indirectly, both Th17 and Th1 cells stimulate epithelial cells to secrete granulopoietic factors and neutrophil chemo-attractants that amplify neutrophil recruitment and activation.

In this study, we clarify the role of T cells and different T cell subsets during autoantibody-induced tissue injury and inflammation and further disentangle the interplay of T cells and neutrophils during this phase. As an example of autoantibody-induced tissue injury, we employ the antibody transfer-induced model of EBA.

## Results

### T cell-deficient mice show an ameliorated clinical phenotype of antibody transfer-induced EBA

**Figure 1:**
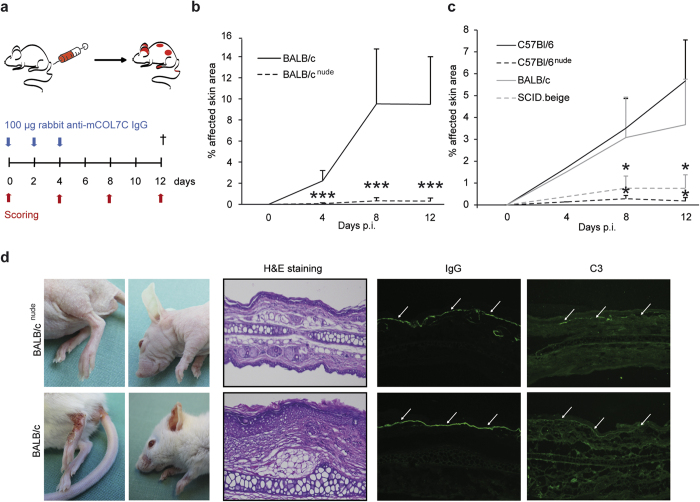
T cell-deficient mice are protected from induction of skin lesions in EBA by antibody transfer. (**a,b**) BALB/c or BALB/c^nude^, (**a,c**) C57BL/6, C57BL/6^nude^ or SCID.beige were injected with 3 × 100 μg affinity-purified rabbit anti-mCOL7 IgG at days 0, 2 and 4. The mean affected body surface area was recorded 4, 8 and 12 days after the first anti-mCOL7 IgG injection (post injection, p.i.) and was reduced in T or T/B cell-deficient mice. These findings suggest a role for T cells during the inflammatory processes in experimental EBA. (**d**) Clinical pictures from BALB/c or BALB/c^nude^ mice at day 12 after injection of affinity-purified rabbit anti-mCOL7 IgG showing a reduced clinical disease severity in nude mice. Histology (H&E staining) indicated reduced inflammation in the epidermis of ear sections of BALB/c^nude^ mice; staining with an anti-rabbit IgG-FITC and anti-mouse C3-fluorescein antibody showed no differences in IgG or C3-deposition at the DEJ. (**b**,**c**) Mann-Whitney-U Test (*p < 0.05, ***p < 0.001), mean (+SD), (**a**) n = 9/group, (**b**) n = 6/group.

To resemble the effector-phase of EBA, we employed the antibody transfer-induced model of EBA: The disease is induced by repetitive injections of affinity-purified rabbit anti-mCOL7 IgG into mice (Fig. 1a). In this model, T cell-deficient BALB/c^nude^ mice were nearly completely protected from EBA induction (Fig. 1b,d). Moreover, depletion of T cells in C57BL/6 wild-type mice using an anti-CD3 antibody showed reduced disease activity between day 6 and day 8 of the experiment (Supplementary Fig. S1). To further exclude strain-dependent effects, EBA was induced in T cell-deficient C57BL/6^nude^ mice, which analogously exhibited a significantly less severe disease score (Fig. 1c). Furthermore, SCID.beige mice, which exhibit impaired B and T cell-development and reduced natural killer (NK) cell activity, showed a similar but less pronounced effect as compared to the nude mice when injected with anti-mCOL7 IgG (Fig. 1c). These effects were independent of IgG- and C3-deposition along the dermal-epidermal junction as shown by direct immunofluorescence (IF) microscopy (Fig. 1d), which remained identical in all strains.

It was necessary to exclude any bias caused by the absence of T cells resulting in compensatory mechanisms by other cell types that may have led to differences in the extent of inflammation; thus, we determined the cell count and activation state of neutrophils because neutrophils are required for blistering and inflammation in experimental EBA^13^. A comparison of differential blood counts of BALB/c and BALB/c^nude^ mice showed no difference in total neutrophil numbers in the strains (Table 1). We next compared the activation status of bone-marrow-derived neutrophils of wild-type and nude mice by (i) the release of reactive oxygen species (ROS) from immune complex (IC)-activated cells (Supplementary Fig. S2a) and (ii) the CD62L-expression of splenic and blood neutrophils (Supplementary Fig. S2b). For both parameters, nude mice showed no reduction in neutrophil activity. Collectively, these data indicate that T cells are involved in the effector phase of EBA.

By contrast to our data, initially developed EBA animal models that involved large amounts of xenogenic rabbit-IgG or high doses of monoclonal antibodies had shown that BALB/c^nude^ mice are susceptible to antibody transfer-induced EBA^9^,^16^,^22^. To confirm the possibility that excessive amounts of xenogenic IgG affect disease progression, we injected 300 μg of affinity-purified rabbit anti-mCOL7 IgG with 30 mg of normal rabbit IgG. As an additional control, mice injected with 30 mg of total anti-mCOL7 IgG were included. The latter led to a disease score in SCID.beige mice, which are comparable to wild-type mice, whereas injection of 300 μg anti-mCOL7 IgG caused nearly no clinical symptoms in SCID.beige mice (Supplementary Fig. S3). Injection of 300 μg affinity-purified rabbit anti-mCOL7 IgG with 30 mg of normal rabbit IgG caused a disease score nearly as high as 30 mg total mCOL7 IgG. This indicates that excessive amounts of xenogenic IgG may promote inflammation through unspecific effects (i.e., activation of myeloid cells by formation of immune complexes), activating multiple pathways that do not include T cells.

### Adoptive transfer of T cells reconstitutes EBA susceptibility in T cell-deficient mice

**Figure 2:**
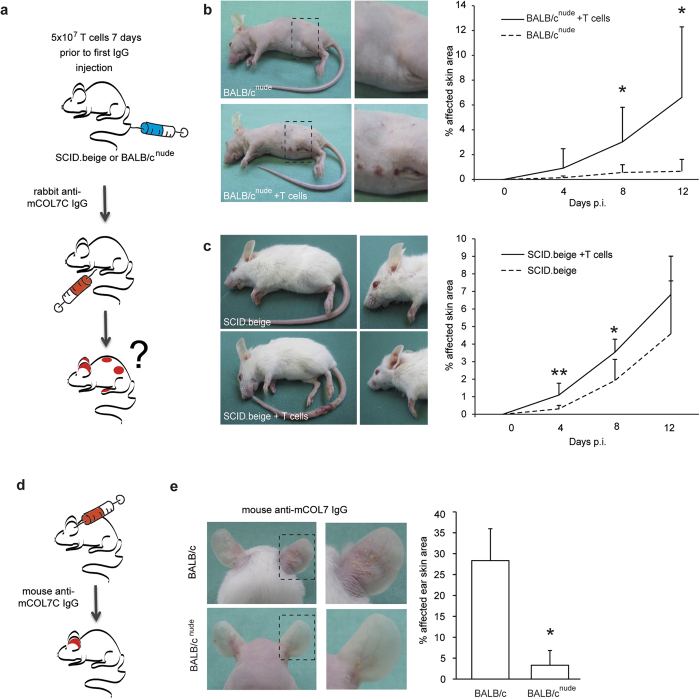
Transfer of T cells reconstitutes susceptibility to skin blistering. (**a**) EBA was induced by repetitive injections of 3 × 100 μg specific rabbit anti-mCOL7 IgG at days 0, 2 and 4. Reconstitution of T cells by i.v. injection of 5 × 10^7^ splenic T cells from BALB/c wild-type mice was performed 7 days prior to the first IgG injection. (**b,c**) T cell reconstitution restored the EBA phenotype after rabbit anti-mCOL7 IgG transfer in BALB/c^nude^ or SCID.beige mice. (**d**) To exclude a functional effect of the production of mouse anti-rabbit IgG, 1 mg mouse anti-mCOL7 IgG or PBS was injected into the ears of either BALB/c or BALB/c^nude^ mice. The clinical picture and mean affected ear surface area was observed 48 h after IgG injection. Again, BALB/c^nude^ mice that were injected with mouse anti-mCOL7 IgG were resistant to EBA induction. (**b,c,e**) Mann-Whitney-U test, respectively (*p < 0.05, **p < 0.01), mean (+SD), (**a**) n = 7/group, (**b**) n = 6/group, (**c**) n = 4/group.

To further validate the contribution of T cells to inflammation and blistering in antibody transfer-induced EBA, BALB/c^nude^ or SCID.beige mice were reconstituted with wild-type T cells. Subsequently, EBA was induced by antibody transfer (Fig. 2a). T cell-reconstitution nearly fully restored the EBA phenotype in BALB/c^nude^ or SCID.beige mice (Fig. 2b,c). To evaluate whether the mice reacted to the injected rabbit IgG, possibly leading to enhancement of inflammation and alternation in the disease phenotype, the presence of anti-rabbit IgG was determined. Anti-rabbit IgG was detected in BALB/c and BALB/c^nude^ mice after reconstitution with BALB/c T cells but not in SCID.beige mice (Supplementary Fig. S4a). To further exclude that the skin inflammation in the reconstituted mice was influenced by the presence of anti-rabbit IgG, a local EBA-model was applied using mouse anti-mCOL7 IgG instead of rabbit IgG for the induction of EBA (Fig. 2d). Within 48 hours of injection of mouse anti-mouse COL7 IgG, BALB/c mice developed inflammation and subepidermal blistering. By contrast, and consistent with the previous findings, BALB/c^nude^ mice were again protected from EBA induction (Fig. 2e). In conclusion, reconstitution of T cell-deficient mice with wildtype T cells restores the clinical EBA-phenotype using the antibody transfer-induced model of EBA, underscoring the relevance of T cells during the effector phase of EBA.

### T cells migrate to the skin after reconstitution in T cell-deficient mice

**Figure 3:**
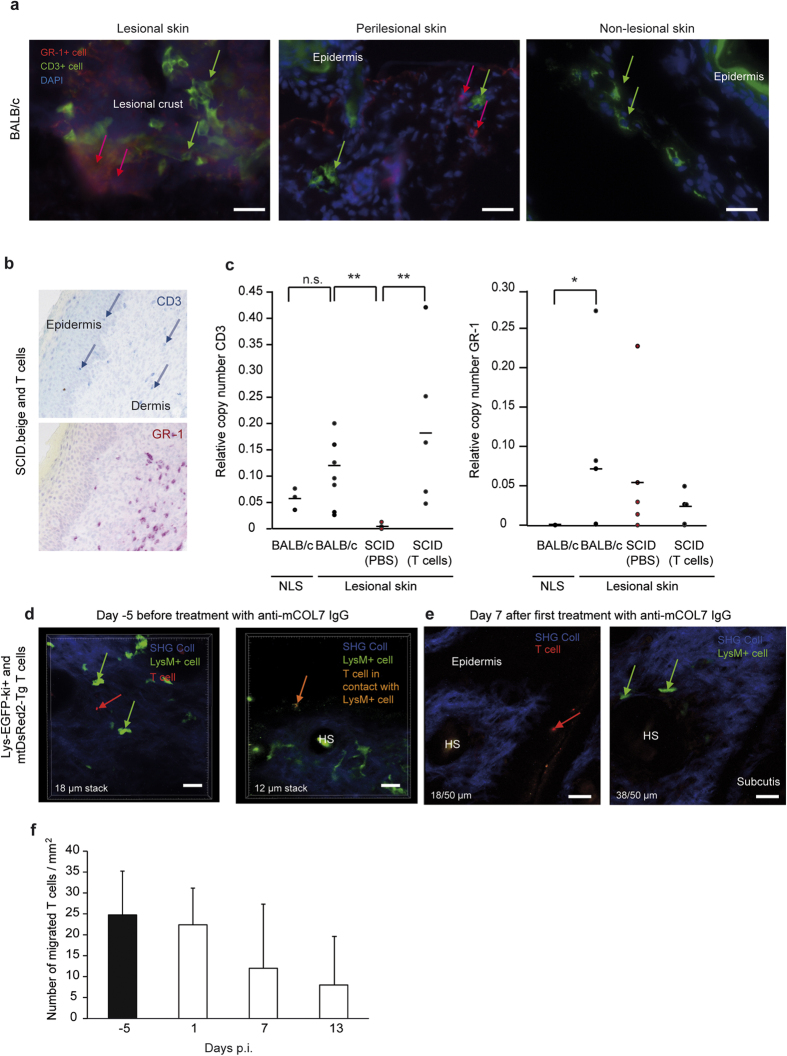
T cells migrate to the skin after reconstitution in T cell-deficient mice. (**a**) To investigate the localization of T cells in the skin after induction of experimental EBA, the disease was induced in BALB/c mice. To analyze T cell expression in the skin, T cell (CD3 + cells, FITC) and polymorphonuclear cell (Gr-1 + cells, DyLight594) staining was performed in BALB/c mice 12 days after the first anti-COL7 injection in the skin. Gr-1 was visible only in lesional and perilesional skin, and CD3 was expressed in all skin specimens. (**b**) To analyze the infiltration of T cells in the skin, SCID.beige mice were reconstituted with T cells by injection of 5 × 10^7^ splenic T cells from BALB/c wild-type mice. Staining for T cells (CD3 + cells, Fast Blue) and polymorphonuclear cells (Gr-1 + cells, Fast Red) was performed in reconstituted SCID.beige mice 12 days after injection with anti-COL7 IgG in the skin. (**c**) EBA was induced in SCID.beige or BALB/c mice. Analysis of mRNA by qRT-PCR for CD3 and Gr-1 was performed in non-lesional skin (NLS) and lesional skin from the same mouse. As expected, Gr-1 was visible only in lesional skin, and CD3 was expressed in reconstituted SCID.beige mice and BALB/c mice to the same extent. Notably, no differences were detectable between lesional and non-lesional skin. (**d,e**) To investigate a functional co-localization of T cells and neutrophils in the skin, mtDsRed2-Tg T cells were injected into lys-EGFP-ki + mice 7 days prior to the injection of rabbit anti-mCOL7 IgG and analyzed on days −5, 1, 7 and 13 using *in vivo* multiphoton microscopy of the ear skin. During all investigated time points, neutrophils were observed exclusively in the dermis, and T cells were observed predominantly in the epidermis. Direct contact between T cells and neutrophils was extremely rare. (**d**) Representative slides of a 50-μm acquired stack show T cells at different layers than neutrophils because of the thickened skin. (**f**) The amount of injected T cells in the ear before/after rabbit anti-mCOL7 IgG injection in lys-EGFP-ki + mice was analyzed in up to eight different acquired stacks per time point and did not change over time. (**c,d**) Two-way ANOVA test with a Bonferroni *post hoc* test (*p < 0.05, p < 0.01), (**f**) n = 8 stacks/group.

To develop deeper insights into the mechanism of T cells and the location in which T cells act during the effector phase of EBA, we evaluated the localization of T cells and their interaction with neutrophils during antibody transfer-induced EBA. The presence and distribution of T cells were analyzed before and during experimental EBA (Fig. 3a). T cells were identified in inflamed areas (defined by the presence of Gr-1 + myeloid cells) as well as in non-inflamed areas of the skin of those mice (Fig. 3a,b). For confirmation, CD3 and Gr-1 mRNA expression was measured in the lesional and non-lesional skin of BALB/c, SCID.beige mice and T cell-reconstituted SCID.beige mice (Fig. 3c).

Next, to test whether T cells and neutrophils co-localize in the skin, T cells were isolated from mtDsRed2-Tg^23^ mice, which express the red fluorescent protein ‘DsRed2′ in their mitochondria. MtDsRed2-Tg T cells were injected into lysozyme M-EGFP-ki (lys-EGFP-ki + ) transgenic mice^24^, in which myelomonocytic cells and particularly neutrophils were labeled with enhanced green fluorescent protein (EGFP). Five days after the T cell injection, EBA was induced via antibody transfer. Using *in vivo* multiphoton microscopy, we followed the dynamics of the migration of T cells and neutrophils to the ear skin of the mice on days −5, 1, 7 and 13 after injection of anti-mCOL7 IgG. Consequently, both neutrophils and T cells migrated to the skin and were detectable on all days of the analysis. At all investigated time points, neutrophils were exclusively observed in the dermis whereas T cells were predominantly located in the epidermis (Fig. 3d,e). Direct contact between T cells and neutrophils was extremely rare. On day 7 after the first rabbit anti-mCOL7 IgG injection, the ears of the mice were inflamed, limiting the detection of cells in the thickened skin (Fig. 3e). Notably, the number of T cells that were observed in the ear did not increase over time (Fig. 3f). All in all, these results indicate that T cells are attracted to skin independently by inflammation.

### Directed migration of T cells and neutrophils is mutually dependent

**Figure 4:**
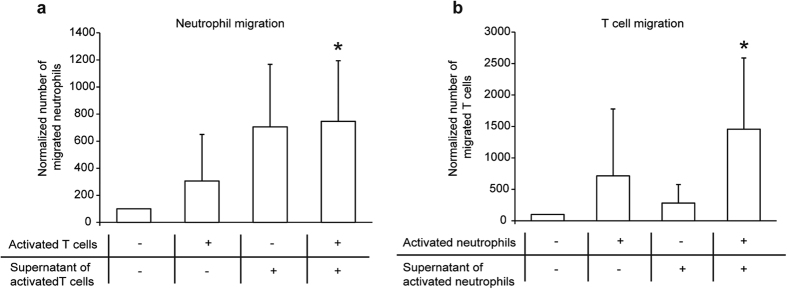
T cell-enhanced inflammation in experimental EBA is not caused by altered neutrophil numbers or activation. (**a**) To analyze whether T cells chemo-attract neutrophils, we performed Transwell™ *in vitro* assays using 3d activated T cells (after stimulation with 1 μm anti-murine CD3/CD28) in the lower and freshly isolated bone marrow derived neutrophils (10^6^ cells per well) in the upper chamber. The number of migrated cells was analyzed by FACS count of GR-1 positive cells. Migration of neutrophils was significantly increased by T cells. (**b**) To analyze conversely whether neutrophil chemo-attracts T cells, we performed Transwell™ *in vitro* assays using 30 min stimulated neutrophil cells (after stimulation with IC) in the lower and freshly isolated splenic T cells (10^6^ cells per well) in the upper chamber. The number of migrated cells was analyzed by a FACS count of CD3 positive cells. Again, migration of T cells was increased by the presence of neutrophils. Two-way ANOVA test with a Bonferroni *post hoc* test (*p < 0.05), mean (+SD), n = 4/group.

To further elucidate the mechanism behind T cell-involvement during the effector phase of EBA, we next hypothesized that certain subsets of T cells attract neutrophils either within the lymphatic organs or to the sites of inflammation in the skin. Therefore, we performed *in vitro* migration assays using activated T cells or IC-activated neutrophils as an attractant for neutrophils and T cells, respectively. Indeed, bone-marrow-derived neutrophils migrated toward anti-CD3/CD28 activated T cells (Fig. 4a). Furthermore, splenic T cells migrated toward neutrophils activated with ICs (Fig. 4b). Because of the dependency of neutrophil migration on IC in these *in vitro* experiments, the data indicate a functional, potentially dual, relevance of the migratory feedback loop also in IC-induced experimental EBA.

### NKT and γδT cells enhance blistering in antibody transfer-induced experimental EBA

**Figure 5:**
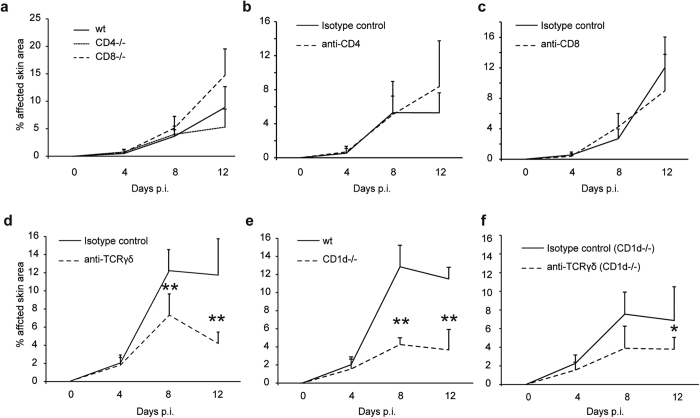
NKT and γδT cells are required for induction of experimental EBA. Mice were injected with 3 × 100 μg specific rabbit anti-mCOL7 IgG at days 0, 2 and 4, and the mean affected body surface area was observed 4, 8 and 12 days after the first rabbit anti-mCOL7 IgG injection. (**a**) CD4−/− or CD8−/− mice have the same disease activity as C57BL/6 mice. (**b**) Depletion with 400 μg/kg CD4 T cell-depleting antibody starting one day prior to IgG injection every second day or (**c**) 5 mg/kg CD8 T cell-depleting antibody at days −1 and 4 revealed no clinical differences. (**d**) Depletion with 4 mg/kg TCRγδ cell-depleting antibody at days −1 and 4 reduced clinical symptoms in BALB/c mice. (e) NKT knockout mice (CD1d−/−) show reduced disease activity, by contrast to BALB/c mice. (**f**) Depletion with 4 mg/kg TCRγδ cell-depleting antibody at days −1 and 4 in CD1d−/− mice further reduced clinical symptoms, leading to the assumption that NKT and γδT cells were the predominant T cell subclasses during experimental EBA. (**a–f**) Mann-Whitney U test (*p < 0.05, **p < 0.01), mean (+SD), (**a**) n = 5/group (**b,c**) n = 8/group, (**d,e,f**) n = 4/group.

Because nude mice are protected from antibody transfer-induced EBA, we first hypothesized that CD4 or CD8 T cells would enhance the clinical phenotype of IC-induced inflammation. Therefore, EBA was induced in CD4 and CD8 knockout mice. Notably, no effect of either CD4 or CD8 deficiency on inflammation and blistering was observed (Fig. 5a). Similar results were obtained in C57BL/6 mice injected with CD4 or CD8 T cell-depleting antibodies (Fig. 5b,c). Next, BALB/c mice were treated with a TCRγδ-depleting antibody. This led to a significant reduction in disease activity, indicating a functional relevance of TCRγδ cells (γδT cells) in mediating inflammation and blistering in IC-induced skin inflammation (Fig. 5d). When experimental EBA was induced in CD1d knockout mice, which exhibit impaired NKT cell functions (Fig. 5e)^25^, a significant reduction of inflammation and blistering was observed. Ultimately, CD1d knockout mice were treated with the TCRγδ-depleting antibody (Fig. 5f), demonstrating a further decrease in inflammation and blistering in the CD1d deficient mice. These experiments demonstrate that NKT cells and γδT cells aggravate the clinical phenotype of antibody transfer-induced EBA.

**Figure 6:**
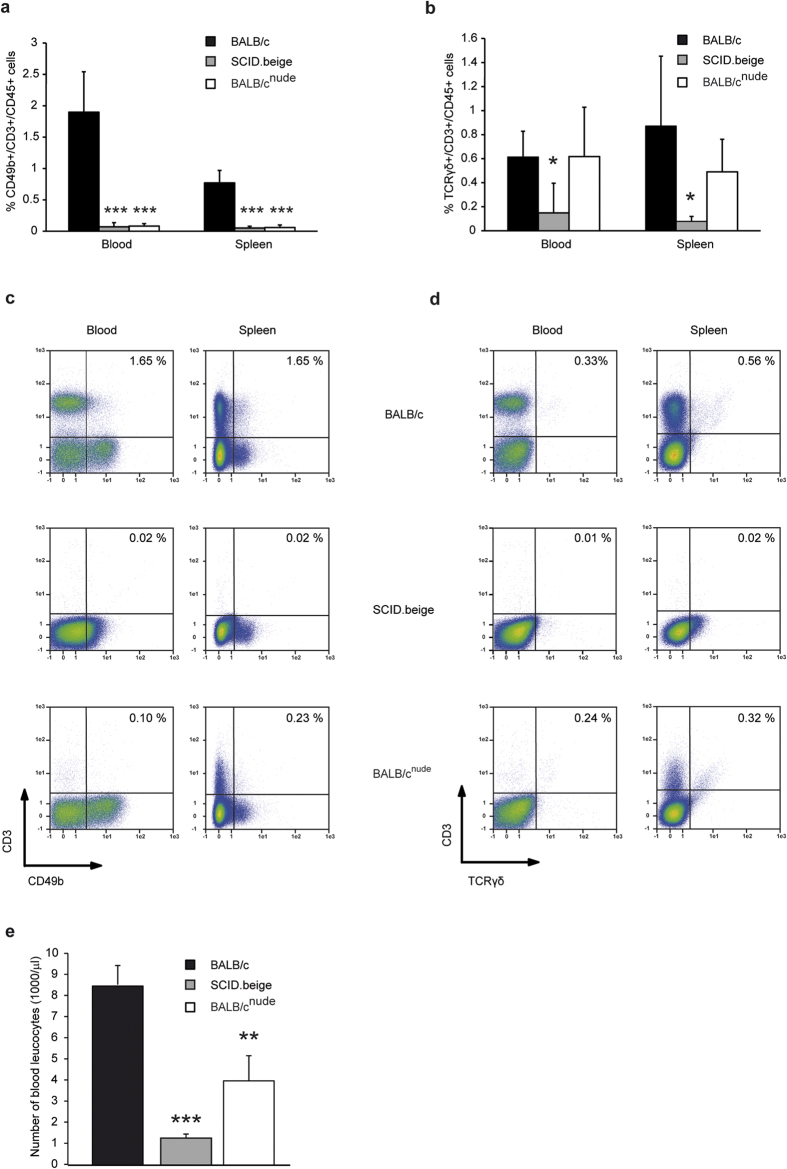
NKT and γδT cells are reduced in T cell-deficient mice. The spleens and blood of T cell-deficient or wildtype mice aged 3 months were analyzed for their content of (**a,c**) NKT cells and (**b,d**) γδT cells. Both SCID.beige and BALB/c^nude^ mice showed scarcely any detectable CD49b+/CD3+/CD45+NKT cells. BALB/c^nude^ mice showed leakiness for CD3 positive cells primarily in the spleen. Because the percentage of TCRγδ+/CD3+/CD45+cells was only reduced in SCID.beige mice (Fig. 6b,d), the total number of leukocytes was analyzed in the blood of T cell-deficient or wildtype mice (Fig. 6e). Indeed, T cell-deficient mice show significantly reduced numbers of total leukocytes, indicating that the total number of γδT cells is also reduced in BALB/c^nude^ mice. (**a,b,e**) 2-way ANOVA with Bonferroni *post hoc* test (*p < 0.05, **p < 0.01, ***p < 0.001), mean (+SD), n = 5/group.

Previous data on NKT cells and γδT cells in BALB/c^nude^ and SCID.beige mice are contradictory. Some studies have reported that extrathymically developed γδT cells can be observed in nude mice^26^. To confirm that BALB/c^nude^ and SCID.beige mice are primarily protected from antibody transfer-induced EBA because of the absence of NKT cells and γδT cells, we investigated the BALB/c^nude^ and SCID.beige mice for the presence of T cells. Both SCID.beige and BALB/c^nude^ mice showed scarcely any detectable CD49b+/CD3+/CD45+NKT cells (Fig. 6a,c). The percentage of TCRγδ+/CD3+/CD45+cells was only reduced in SCID.beige but not in BALB/c.nude mice (Fig. 6b,d). However, the total number of leukocytes was significantly reduced in these strains (Fig. 6e), indicating that the total number of γδT cells was also reduced in BALB/c^nude^ mice.

To further confirm that during the above experiments, we had reconstituted BALB/c^nude^ and SCID.beige mice with NKT cells and γδT cells, we also evaluated for the proportions of these cell types among the T cells that were injected. The results indicated that 1.27% of the cells were γδT cells and at least 3.09% of the cells were NKT cells (Supplementary Fig. S4b). Considering the absolute numbers, this corresponds to 6.4 × 10^5^ γδT cells and 1.6 × 10^6^ NKT cells per injection.

To verify a direct effect of TCRγδ or NKT cells on inflammatory cells like neutrophils in more detail we then isolated these cells from murine spleens and restimulated the cells for 18 h. The cultured cells, the supernatant or the combination of both was subsequently co-cultured with freshly isolated murine neutrophils. The neutrophils were stained for cells surface markers to evaluate the activation status. Indeed, TCRγδ cells could directly affect the activation status of neutrophils. First, the expression of CD18 and the CD62L shedding is significantly increased after co-culture of neutrophils and TCRγδ cells (Supplementary Fig. S5a). Second, the pro-inflammatory cytokines TNF-alpha and IL-6 were significantly higher in the co-culture then in cultured TCRγδ or neutrophils (Supplementary Fig. S5b). The effects of NKT cells on neutrophils were analyzed in the same manner. Anyway, only the release of TNF-alpha was increased in co-culture of NKT cells and neutrophils (Supplementary Fig. S5c), indicating a more prominent effect by TCRγδ than NKT cells on the activation of inflammatory cells like neutrophils.

## Discussion

Our data provide evidence of a pro-inflammatory role of NKT cells and γδT cells during the effector phase of EBA, an antibody-driven and neutrophil-dependent autoimmune inflammation. We here hypothesize that T cells may tip the balance towards inflammation during the effector phases of autoantibody-induced tissue inflammation.

Previous data showed that NKT cells and γδT cells are involved in inflammatory processes in the skin, able to enhance or dampen inflammation. For example, NKT cells can suppress immune responses during various conditions such as cancer or UV-induced photo damage. Conversely, NKT cells are also able to activate immune responses, such as in psoriasis and liver inflammation^27^,^28^,^29^. In this paper we demonstrate that other T cell subclasses are not involved in the effector phase of our animal model of EBA. This notion, however, must be interpreted with care: Different subtypes of CD4- and CD8 T cells may act as pro-inflammatory factors whereas others, such as regulatory T cells, may retard inflammation. Furthermore, in antibody transfer-induced EBA, the onset of inflammation is rapid, occurring within a few days. Unlike in human EBA-patients^30^,^31^, there are no COL7-specific T cells in antibody-transfer induced EBA^30^,^31^,^32^. However, recent data from other AIBD models indicated that antigen-specific T cells can augment blistering and induce inflammation independent of the presence of autoantibodies^33^. Therefore, in the antigen-independent modulation of autoantibody-induced inflammation, antigen-specific T cells may be a third pillar mediating inflammation.

Whether T cells contribute to autoantibody-induced skin inflammation had been unclear. This study demonstrates an involvement of γδ and NKT cells. Based on the current understanding of EBA pathogenesis, the effector phase of EBA, i.e., autoantibody-induced tissue injury, is driven by myeloid cells, i.e., neutrophils: After the binding of autoantibodies to COL7, the complement system is activated and cytokines are released. This collectively leads to a pro-inflammatory milieu that in turn promotes the extravasation of myeloid cells into the skin. Myeloid cells bind to the IC in an Fc receptor-dependent manner, leading to their activation. In EBA, myeloid cell-activation in the skin leads to the release of ROS and proteolytic enzymes that cause blister formation^6^. Based on the data presented here, we propose an additional, novel pathway that primarily involves γδT cells and NKT cells: After the formation of the tissue-bound IC in the target organ, NKT cells and γδT cells that are present in the skin become activated. Possibly three non-mutually exclusive mechanisms may lead to their activation: (i) γδT cells and NKT cells can be observed in the skin^34^,^35^ and modulate inflammatory processes^35^,^36^,^37^. Those cells may either directly contribute to inflammation^38^ or activate and, according to our results, attract neutrophils into the skin^39^. (ii) T cells activate and lead to a disturbed function of keratinocytes by the release of pro-inflammatory mediators^40^,^41^,^42^. Regarding this matter, γδT cells have been shown to induce keratinocyte hyperproliferation via secretion of IL-22 whereas NKT cells activate keratinocytes via IFN-γ^39^. (iii) γδT cells and NKT interact with other cell types, possibly neutrophils, in the lymphatic organs^43^. Previous studies have shown that neutrophils are important regulators of NK cell homeostasis and activation in both mice and humans^44^. A possible mechanism would be that neutrophils prime naïve T cells^45^, which then migrate to the skin and contribute to inflammation as previously discussed. The third hypothesis is consistent with our data which showed that T cells chemoattract neutrophils and neutrophils chemoattract T cells vice versa. To figure out the exact mechanism by which γδT or NKT cells influence neutrophils we co-cultured these cells and showed an increase of neutrophil activation indicated by increased CD18 expression and CD62L shedding during co-culture. Furthermore, we could show that the release of pro-inflammatory cytokines like TNF-alpha is increased in co-culture of neutrophils with γδT cells or NKT cells. These data indicate a direct effect of γδT and NKT cells on neutrophils, nevertheless additional mechanisms cannot be excluded.

In conclusion, we clarify the contradictory findings concerning the *in vivo* and *in vitro* data regarding T cell involvement in the effector phase of IC-driven inflammation, exemplified by EBA. We identify NKT and γδT cells as cells with as yet unrecognized modulatory functions to enhance IC-induced, myeloid cell-dependent inflammation. The effects of NKT and γδT cells in driving inflammation are not absolute; however, they may tip the balance toward inflammation. This result may also explain presence of inflammation and blistering at specific sites on the skin, despite an equal distribution of IC along the entire dermal-epidermal junction. Thus, modulation of NKT and γδT cells may be a promising and novel therapeutic target in IC-mediated autoimmunity.

## Material and Methods

### Mice

BALB/cJ, C57Bl/6 J, BALB/c^nude^ (CAnN.Cg-Foxn1nu/Crl), SCID.beige (CB17.Cg-PrkdcscidLystbg-J/Crl) and SJL/J mice were obtained from Charles River Laboratories (Sulzfeld, Germany). C57Bl/6nude (B6.Cg-Foxn1nu/J), CD4-deficient (ko) (B6.129S2-Cd4tm1Mak/J), CD8-ko (B6.129S2-Cd8atm1Mak/J), CD1d ko (C.129S2-Cd1tm1Gru/J) and DO11.10 (C.Cg-Tg(DO11.10)10Dlo/J) mice were purchased from the Jackson Laboratory (Bar Harbor, Maine, USA). Lys-EGFP-ki + mice were kindly provided by Dr. Graf, Hamburg^24^, and mtDsRed2-Tg mice were kindly provided by Dr. Shitara, Tokyo^23^. Gender-matched mice aged 6 to 12 weeks were used for the experiments. The mice were fed with standardized mouse chow and acidified drinking water *ad libitum*. All clinical examinations, biopsies and bleedings were performed under anesthesia using intraperitoneal (i.p.) administration of a mixture of ketamine (100 mg/g, Sigma-Aldrich, Taufkirchen, Germany) and xylazine (15 mg/g, Sigma-Aldrich) if not stated otherwise. All animal experiments were conducted according to the European Community rules for animal care, approved by the respective governmental administration (V 242.7224.122-5, V312-7224.122-5, V242-7224.122-5. V312-7224.122-5, Ministry for Energy, Agriculture, the Environment and Rural Areas), and performed by certified personnel.

### Generation of anti-mouse COL7 IgG

Total rabbit anti-murine COL7 IgG (total rabbit anti-mCOL7 IgG) was prepared as described^22^. Rabbits were immunized with recombinant proteins of the non-collagenous (NC)−1 domain of murine COL7 by a commercial supplier (Eurogentec, Köln, Germany). IgG from immune and normal rabbit sera were purified by affinity chromatography using protein G. For isolation of mCol7C-specific IgG (rabbit anti-mCOL7), a second affinity chromatography using mCOL7C-coupled Affi-Gel 10 (Bio-Rad, München, Germany) was performed using the manufacturer’s protocol with minor modifications. Briefly, 5 mg/ml mCOL7 in MOPS buffer (0.1 M MOPS, 80 mM CaCl2, pH7.5) was incubated for 1 h with MOPS buffered Affi-Gel 10 at RT. After incubation with MOPS buffer/ethanolamine (1:20) for 1 h, the mCOL7-coupled gel was washed with PBS and was ready for use. To isolate the mCOL7-specific IgG, the isolated rabbit anti-murine COL7 IgG from the first affinity chromatography was incubated on the mCOL7C-coupled Affi-Gel 10 for 1 h at 4 °C with gentle shaking. After washing with PBS, the column was washed with washing buffer (850 nM NaCl, 0.1% Triton-X-100) until the unspecific IgG was completely washed away (OD280 < 0.01). For elution, the column was flushed with elution buffer (0.1 M glycine, pH 2.8). The eluate was neutralized to pH 7.2 using Tris-buffer and concentrated using an Amicon Ultra-15 filter (Millipore, Darmstadt, Germany). Normal rabbit (NR) IgG was used as a negative control. Mouse anti-murine COL7 IgG was prepared as described^5^,^46^. In brief, SJL/J mice were immunized with 60 μg recombinant mCOL7^46^. IgG from immune mouse serum (mouse anti-mCOL7) was purified by affinity chromatography using protein G affinity (GE Healthcare Biosciences, Berlin, Germany) as described previously^5^. Reactivity of all IgG fractions was analyzed by immunofluorescence microscopy on murine skin.

### Induction of experimental EBA

Antibody-induced transfer studies for the induction of experimental EBA followed published protocols with minor modifications^5^. If not otherwise noted, mice received a total of three i.p. injections of 100 μg of specific rabbit anti-mCOL7 IgG or corresponding amounts of normal rabbit IgG every second day. Higher doses of rabbit anti-mCOL7 IgG (6 times 100 μg) or a total of 6 i.p. injections of 5 mg of total rabbit anti-mCOL7 IgG (without purification using mCOL7 domain mCOL7C^9^ coupled Affi-10 columns) was used for the respective experiments indicated in the text. Disease severity was expressed as the percentage of body surface area affected by skin lesions determined at four time points (days 0, 4, 8 and 12). Different body parts were individually scored by the appearance of crust, erythema, lesions and/or alopecia. The total body score is composite of 2.5% per ear, snout and oral mucosa; 0.5% per eye; 9% for head and neck (excluding eyes, ears, oral mucosa and snout), 5% per front limb, 10% per hind limb and 40% for the remaining trunk. Blood and tissue samples were collected on day 12. All experiments were repeated in a minimum of two independent experiments using different batches of purified rabbit anti-mCOL7 and NR IgG. This antibody-transfer EBA duplicates the clinical, histological, and immunopathological findings of the inflammatory variant of the human disease^9^. Local experimental EBA was induced by injecting 1 mg (50 μl in PBS) of mouse anti-murine COL7 IgG into the mouse ear base^5^. After 48 h, development of crust and erosions in the injected ear were visible, and the percentage of affected ear skin area was determined. As a control, PBS was injected into the contralateral ear.

### Treatments with anti-CD3, anti-CD4, anti-CD8, anti-TCRγδ and T cell-injections

Hamster anti-mouse CD3 monoclonal antibody (clone: 145-C11) and LEAF purified anti-mouse TCRγδ (clone: UC7-13D5) were obtained from BioLegend, Fell, Germany. Mice were treated i.p. with 10 μg anti-CD3 or 100 μg anti- TCRγδ 1 day prior to and 4 days following the first IgG injection. Rat anti-mouse CD4 and CD8 monoclonal antibodies (mAbs) were obtained from the hybridoma cell lines GK1.5 or 53-6.72, respectively. Hybridoma cell lines were grown in RPMI 1640 medium (Life Technologies, Darmstadt, Germany) supplemented with 10% heat-inactivated IgG free FCS (PAA Laboratories, Pasching, Austria), 100 U/ml Pen-Strep (PAA Laboratories), 2 mM L-glutamine, and 50 μM 2-ME (Sigma-Aldrich). Supernatants were purified over a protein G column (GE Healthcare Biosciences, Berlin, Germany), dialyzed in PBS, and concentrated in polyethylene glycol 20,000 (Merck, Darmstadt, Germany)^5^. One day before and 1, 3, 5, 7, 9, and 11 days after the first IgG injection, mice were injected i.p. with 400 μg/kg body weight of the anti-mouse CD4 mAb. One day before and 4 days after the first IgG injection, mice were injected i.p. with 5 mg/kg body weight of the anti-mouse CD8 mAb. For all injections, an isotype control antibody in the respective concentration (either rat IgG or clone HTK888) served as a control. In a pilot experiment, the ability to deplete the respective cell populations in C57BL/6 J mice was confirmed by flow cytometry (FACS) analysis of peripheral blood cells from treated mice. For this purpose, a 50 μl-blood sample was taken from the tail vein with 25 μl of 20 mM EDTA. T cells were isolated from murine spleens using a PanT cell isolation kit II (Miltenyi, Bergisch-Gladbach, Germany), following the manufacturer’s protocol. A total of 5 × 10^7^ cells (in PBS, injection volume 100 μl) or 100 μl PBS as control were injected i.v. 7 days prior to the first IgG injection. T cells were isolated from either BALB/c or DO11.10 mice or (for 2-photon microscopy) from mtDsRed2-Tg mice^24^.

### Multiphoton microscopy

T cells from mtDsRed2-Tg mice (DsRed + T cells) were injected i.v. 7 days prior to the first IgG injection into lys-EGFP-ki + mice^24^. In lys-EGFP-ki + mice, events leading to inflammation and blistering in antibody transfer-induced EBA can be investigated, i.e., the migration behavior of lys-EGFP + neutrophils and monocytes (unpublished). Lys-EGFP-ki + mice were anesthetized via injection for long-term experiments (mixture of 50 μl Fentanyl, 400 μl Midazolam, 200 μl Dormitor and 3.5 ml Ringer solution). Next, hair removal cream (GlaxoSmithKline, Bühl, Germany) was applied to the ears to remove the autofluorescent hair shafts. Imaging was performed using a TriM Scope two-photon microscope (LaVision BioTec GmbH, Bielefeld, Germany) equipped with a XLUMPLFL 20 × W/0.95 objective (Olympus, Hamburg, Germany)^47^. The excitation wavelength for the visualization of DsRed was 740 nm (T cells). EGFP (neutrophils and macrophages) and the second harmonic generation signal for type I collagen were excited with 940 nm. Emitted light was detected by three wavelength-separated PMTs (435–495 nm, 495–560 nm and >560 nm). Visualization of the neutrophil and T cell migratory behavior in the skin was performed on days −5, 1, 7 and 13 after the first anti-COL7 IgG injection for the induction of experimental EBA. As many as eight different Z-stacks were acquired at each time point of the experiment. Each stack was the size of 250 × 250 × 12 to 100 μm. Image processing was conducted using Imaris Software (Bitplane, Zurich, Switzerland), and the total number of extravasated T cells was counted by hand.

### Immunofluorescence (IF), immunohistochemistry and histological studies

Direct IF microscopy to detect rabbit IgG and murine C3 in experimental EBA was performed as described^48^. Briefly, frozen sections were prepared from tissue biopsies and incubated with goat anti-rabbit antibodies reactive with rabbit IgG (Dako Deutschland GmbH, Hamburg, Germany) and murine C3 (MP Biomedicals LLC, Keyseberg, France). Both sections were labeled with fluorescein isothiocyanate (FITC). For immunofluorescence, cryostat sections from lesional, perilesional and non-lesional skin were stained for T cells and granulocytes. Briefly, biotinylated antibodies against CD3 (clone 145-2C11, BioLegend) and an anti-Gr-1 antibody (Ly6G, RBC-8C5, eBioscience, Germany) were used as primary antibodies, and either DyLight594 goat anti-rat IgG (Dianova, Hamburg, Germany) or Streptavidin-FITC (Thermo Fisher Scientific, Waltham, USA) was the secondary Ab. Nuclei were counterstained using DAPI (Sigma-Aldrich). For immunohistochemistry, cryostat sections from lesional and non-lesional skin were stained for T cells and granulocytes as described earlier^49^. Briefly, mAbs against TCRb (BD Biosciences, Heidelberg, Germany) and Gr-1 (Ly6G, RBC-8C5, eBioscience) were used as primary antibodies, and either alkaline phosphatase goat anti-rat IgG (Roth, Karlsruhe, Germany) or goat anti-hamster (Abcam, Berlin, Germany) was used as secondary Ab. Alkaline phosphate activity was visualized with Fast Blue (BB Salt, Sigma-Aldrich). Samples were stained by hematoxylin according to standard protocols. For histology, skin samples were fixed in 3.7% paraformaldehyde. The 8 μm-thick sections from paraffin-embedded tissues were stained with H&E.

### Differential blood count and flow cytometry

Differential blood counts were performed using an automated veterinary hematologic analyzer with a preprogrammed murine calibration mode (Hemavet 950FS, Drew Scientific) according to the manufacturer’s instructions. For FACS analysis, the following antibodies were used: Vio-Green, Brilliant Violet (BV) 421™, FITC-, phycoerythrin (PE)- or allophycocyanin (APC)-conjugated anti-mouse CD3 (clone 145-2C11), CD4 (clone L3T4, or RM4-5), CD8 (clone 53–6.7), CD49b (clone DX5), TCR γ/δ (clone GL3), CD45 (clone: 30F11) Gr-1 (clone RB6-8C5), or appropriate isotype control antibodies (eBioscience, Miltenyi or BD). Cell suspensions were blocked with anti-mouse CD16/CD32 mAb before staining, and dead cells were excluded from the analysis using propidium iodide (PI) 56. Briefly, for the staining of NKT and TCRγδ cells, the erythrocyte lysed and blocked single cell suspensions from spleen, mesenteric lymph node and blood were stained for PI, CD45-VioGreen, TCR γ/δ-BV421, CD49b-APC and CD3-PE. Cells were first gated for singlets (FSC-H compared with FSC-A) and leukocytes (SSC-A compared with FSC-A). The leukocyte gates were further analyzed for their uptake of PI to differentiate live from dead cells and their expression of CD45, taking only the live, healthy leukocyte population. For analyzing the purity of isolated CD3 cells before reconstitution, cells were stained with CD3-FITC and PI only. The purity of cells was between 94% and 97%; viability was more than 90%. For validation of cell depletion after treatments with anti-CD3, anti-CD4, anti-CD8 antibodies, anti-TCRγδ and either CD3, CD4, CD8 or CD3/TCR γ/δ were used for the staining of blood samples before and after the depletion (data not shown). To determine the activation status of neutrophils in the spleen in wild-type compared with T cell depleted mice, erythrocyte lysed spleen cells were stained with CD62L-FITC, Gr-1-APC and PI; only FACS analysis was performed at the Miltenyi MacsQuant10 or FACSCalibur and analyzed with the MACSQuantify™ Software (Version 2.6).

### Assessment of neutrophil activation by ROS

Neutrophil activation was assessed by the determination of IC-induced ROS release using a previously published protocol^50^ with minor modifications. In brief, the mouse femurs were isolated, and the cells were washed out with 10 ml PBS using a 26 G syringe. Neutrophils were isolated using Percoll gradient centrifugation (GE Healthcare, Germany) following the protocol provided by the manufacturer and a negative selection for CD3 and CD19 using CD19-PE and CD3-PE primary antibodies and PE-coupled beads from Miltenyi, Germany following the manufacturer’s protocol. Isolated neutrophils (10^5^ cells/200 μl) were incubated on a 96-well plate (Greiner Bio-One, Frickenhausen, Germany) coated with IC formed by mCOL7 and rabbit anti-mCOL7 IgG. ROS release was analyzed using luminol (Sigma-Aldrich). Each plate was analyzed for 60 repeats using a plate reader (PerkinElmer, San Jose, CA, USA), and the values are expressed as relative fluorescence units.

### Assessment of neutrophil activation by analysis of cell surface markers and cytokine release

TCRγδ or NKT cells were isolated from murine spleens using the TCRγ/δ + T Cell Isolation Kit (using BALB/cJ mice) or the NK1.1 + iNKT Cell Isolation Kit (using C57BL/6 J mice), respectively (Miltenyi), following the manufacturer’s protocol. The enrichment of cells was determined by FACS. Isolated TCRγδ cells were 78% positive by staining with CD3-PE/TCRγδ−BV421 (Biolegend), isolated NKT cells had 77% positive staining using CD3-PE/CD49b-APC (Biolegend). Isolated TCRγδ or NKT cells were restimulated for 18 h using 5 μg/ml anti mCD3 (Biolegend, clone 145-2C11). The cultured cells, the supernatant or the combination of both was subsequently co-cultured with freshly isolated murine neutrophils^50^ in a ratio of 1:4 for additional 4 h. The neutrophils were stained for FACS using CD18-FITC, CD62L-PE-Vio770, Ly6G-APC-Vio770 and CD45-VioGreen (Miltenyi) following standard procedures to evaluate the activation status. Dead cells were excluded using PI. The supernatant of cultured cells was analyzed for cytokine release using the LEGENDplex™ Mouse Proinflammatory Chemokine Panel (Biolegend).

### ELISA for detection of circulating mouse anti-rabbit IgG

Serum levels of circulating mouse anti-rabbit IgG were determined by ELISA using mouse IgG quantification sets (Bethyl, Montgomery, Texas, USA). In detail, each well was coated with 250 ng normal rabbit IgG. After blocking, a series of diluted samples was added and incubated for 60 min. Bound Abs were detected by HRP-conjugated goat anti-mouse IgG (Bethyl) and tetramethylbenzidine (Thermo Fisher Scientific). The enzymatic color reaction was stopped by 2 M sulfuric acid (Carl Roth, Karlsruhe, Germany), and the change in OD was measured with a plate reader (PerkinElmer, Waltham, Mass., USA) at 450 nm. Standard reference curves were established using the provided mouse reference sera (Bethyl).

### Transwell migration assay

#### Neutrophil migration

Murine T cells (3 × 10^5^ cells/well) were cultured over 3 days at 37 °C in migration medium (RPMI 1640 medium [Life Technologies, Darmstadt, Germany]) supplemented by 10% heat-inactivated IgG free FCS (PAA Laboratories, Pasching, Austria), 100 U/ml Pen-Strep (PAA Laboratories), 2 mM l-glutamine and 5 μg/ml anti-mCD28 (Biolegend, clone 37.51) in the bottom chambers of 24-well transwell plates (pore size 5 μm) coated with 5 μg/ml anti mCD3 (Biolegend, clone 145-2C11). After 3 days, the supernatant of the T cells was either left inside the well or transferred into a separate well for analysis. The upper chambers were filled with freshly isolated neutrophils (10^6^ cells/well) in migration medium, and the entire plate was incubated for 45 min at 37 °C for migration.

#### T cell-migration

Murine neutrophils (10^6^ cells/well) were activated for 30 min in the bottom chambers of 24-well transwell plates (Corning Costar, Sigma, pore size 5 μm) coated with mCOL7/rabbit anti-mCOL7 IgG IC. Thereafter, the supernatant of the neutrophils was either left inside the well or transferred into a separate well for analysis. The upper chambers were filled with freshly isolated T cells (10^6^ cells/well) in migration medium, and the entire plate was incubated for 45 min at 37 °C for migration.

#### Determination of cell number

After migration, the cells in the lower chamber were harvested, and the number of cells was evaluated using FACS analysis (anti-mGR-1-PE, anti-mCD3-FITC) to determine the percentage of T cells and neutrophils; the Neubauer chamber was used to calculate the total number of cells. A minimum of three independent experiments was performed, and the number of cells migrating without stimulus was normalized to 100.

### Statistical analysis

Data were analyzed using SigmaPlot, version 12 (Systat Software Inc., Chicago, USA). Applied tests and confidence intervals are indicated in the respective text and figure legends. A p-value < 0.05 was considered statistically significant.

## Additional Information

**How to cite this article**: Bieber, K. *et al*. T cells mediate autoantibody-induced cutaneous inflammation and blistering in epidermolysis bullosa acquisita. *Sci. Rep.* **6**, 38357; doi: 10.1038/srep38357 (2016).

**Publisher's note:** Springer Nature remains neutral with regard to jurisdictional claims in published maps and institutional affiliations.

## Supplementary Material

Supplementary Data

## Figures and Tables

**Table 1:** Differential blood counts of BALB/c or BALB/c^nude^ mice.

	**BALB/c**^**nude**^	**BALB/c**	**p-value**
Total leukocytes	4.00 ± 1.20	8.49 ± 0.90	0.0066
Neutrophils	1.23 ± 0.73	0.95 ± 0.13	n.s.
Lymphocytes	2.45 ± 1.19	7.20 ± 0.76	0.0043
Monocytes	0.30 ± 0.34	0.34 ± 0.03	n.s.
Eosinophils	0.02 ± 0.01	0.00 ± 0.01	n.s.
Basophils	0.00 ± 0.01	0.00 ± 0.00	n.s.
Erythrocytes	8104.17 ± 1066.71	8420.83 ± 256.58	n.s.
Platelets	481.67 ± 94.03	435.42 ± 29.62	n.s.

Data presented are based on n = 3 per group. Comparisons between groups were performed using Mann-Whitney-U test (n.s. = not significant), n = 5/group.
