# Supplementary material for: T cells mediate autoantibody-induced cutaneous inflammation and blistering in epidermolysis bullosa acquisita
Source: Sci Rep. 2016 Dec 5;6:38357. doi: 10.1038/srep38357 (PMC5137106; doi:10.1038/srep38357)
Supplement: Supplementary Data [file srep38357-s1.pdf]

Supplementary Data to

‚T cells mediate autoantibody-induced cutaneous inflammation and blistering in epidermolysis bullosa acquisita´

by Katja Bieber, Mareike Witte, Shijie Sun, Jennifer E. Hundt, Kathrin Kalies, Sören Dräger, Anika Kasprick, Trix Twelkmeyer, Rudolf A. Manz, Peter König, Jörg Köhl, Detlef Zillikens and Ralf J. Ludwig

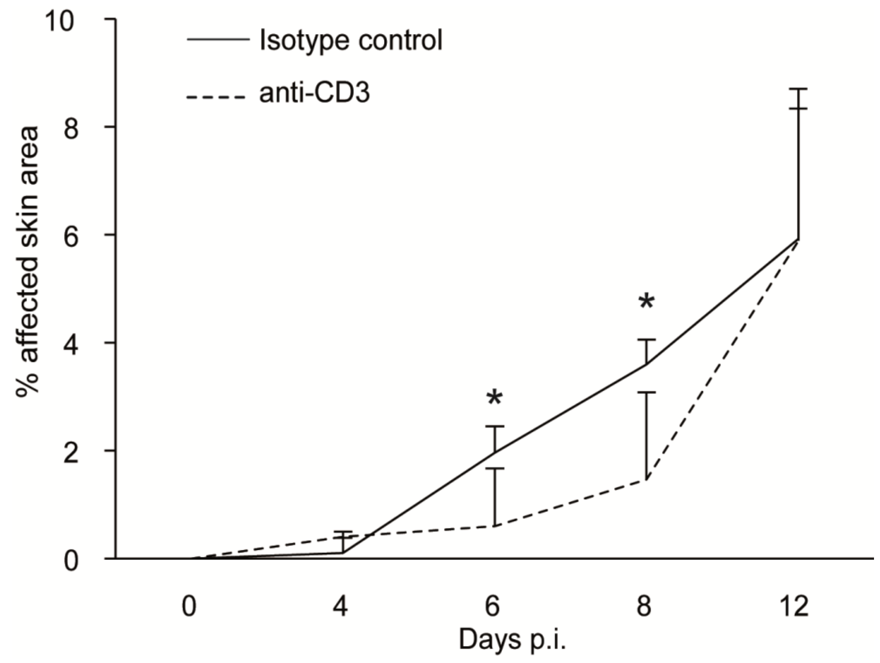

Supplementary Figure S1. Inhibition of T cells in experimental EBA reduces disease severity. To confirm the T cell dependency in experimental EBA, C57BL/6 mice were injected with 3x100 µg affinity-purified anti-mCOL7 IgG at days 0, 2 and 4, and the mean affected body surface area was assessed. T cells were depleted using 10 µg of an anti-CD3 antibody (clone 145-C11) at days -1 and 4. Again, mice with no/lower T cells showed a reduced clinical EBA phenotype. Mann-Whitney-U test (*p<0.05), mean (+SD), n = 7.

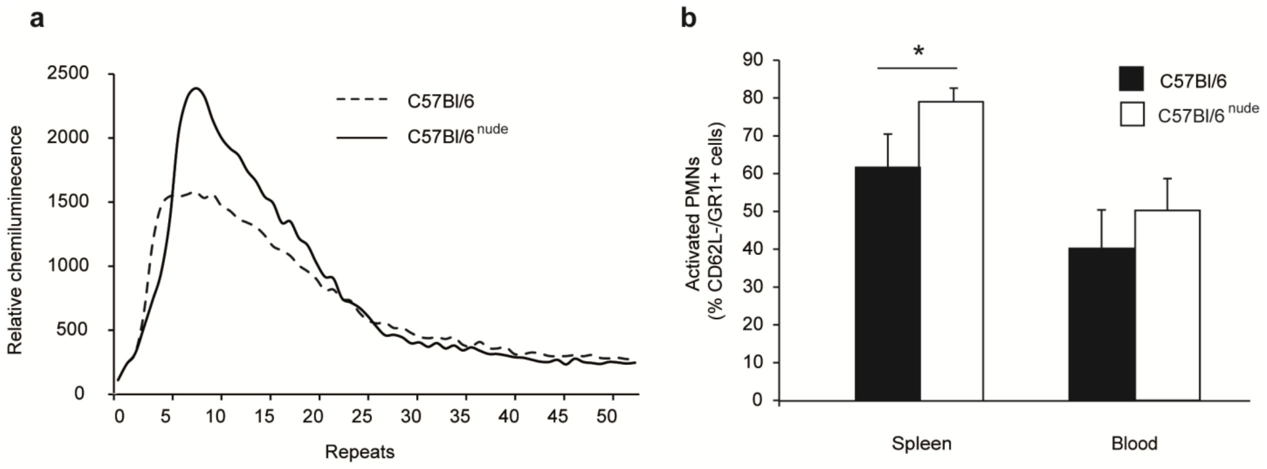

Supplementary Figure S2. Neutrophil activation is not enhanced in T cell-deficient mice. (a) The activation status of bone marrow derived neutrophils from wild-type and nude mice was analyzed in vitro by measuring the release of reactive oxygen species (ROS) from immune complex-activated cells. Here, one representative result of three independent experiments is shown. The average cumulative ROS release, expressed as the area under the curve, amounted to 32,906 in wild-type and 25,975 in nude mice. (b) Next, the expression of CD62L on splenic and peripheral Gr-1+ cells was analyzed by flow cytometry. We observed a higher frequency of CD62L negative cells in splenic Gr-1+ cells in nude mice than in wild-type mice, showing a higher state of neutrophil-activation in these animals. No difference in CD62L expression was noted in circulating Gr-1+ cells. (b) Mann-Whitney-U test (*p<0.05), mean (+SD), (a) n = 5, (b) n=4.

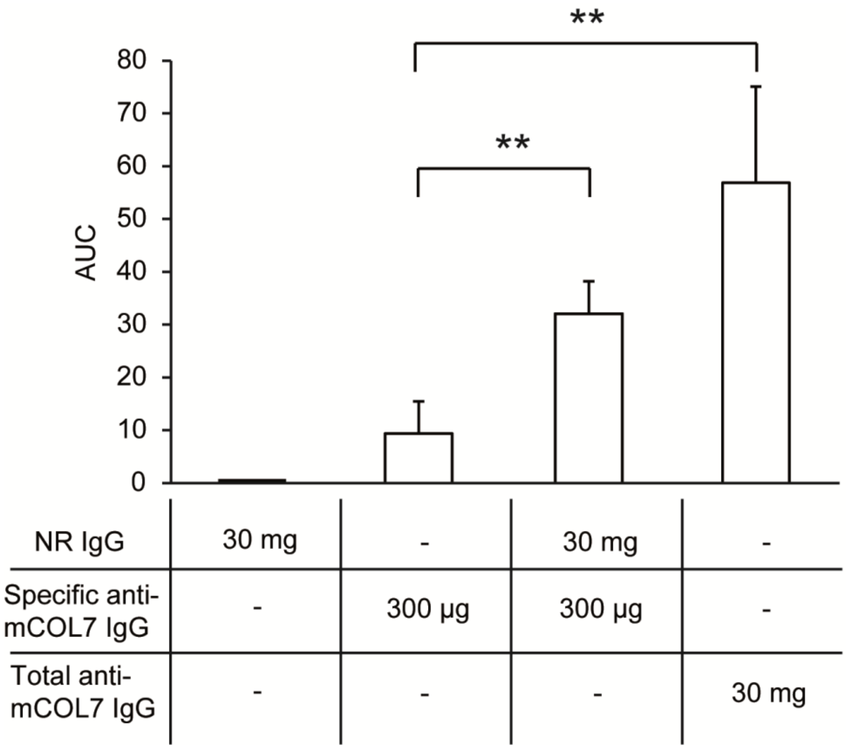

Supplementary Figure S3. Addition of high amounts of xenogenic antibodies increases disease severity in T cell-deficient mice. To examine the relevance of xenogenic antibodies to disease progression, SCID.beige mice were injected with either 6x50 µg specific rabbit anti-mCOL7 IgG (specific mCOL7 IgG), 6x5 mg total rabbit anti-mCOL7 IgG (total mCOL7 IgG) or normal rabbit IgG (NR IgG) from rabbit serum or a combination of 6x50 µg specific rabbit anti-mCOL7 IgG and NR IgG at days 0, 1, 2, 3, 4 and 5; and the area under curve (AUC) from days 1-12 post injection (p.i.) was calculated. Notably, SCID.beige mice showed increased disease severity when they were injected with a combination of 300 µg specific rabbit anti-mCOL7 IgG and 30 mg NR IgG. Two-way ANOVA test with Bonferroni post hoc test (**p<0.01), mean (+SD), n = 4 (NR-IgG), n=5 (other experimental groups).

a

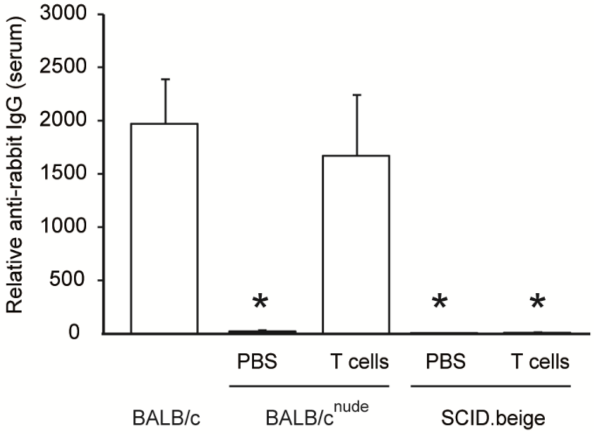

b

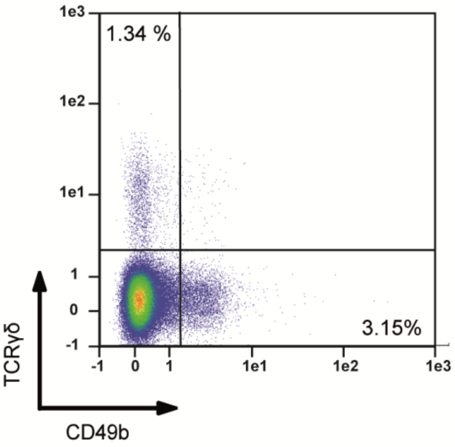

CD3/TCRγδ	1.27 ± 0.09
CD3/CD49b	3.09 ± 0.67

Supplementary Figure S4. Analysis of the quality of transferred T cells. (a) To confirm that the transferred T cells were able to activate B-cells, we analyzed the production of a mouse anti-rabbit antibody in the T cell-deficient mice that were reconstituted with wildtype T cells. Production of mouse anti-rabbit antibodies 12 days after the first IgG injection was analyzed by ELISA in BALB/c (without T cell-reconstitution) as well as BALB/cnude and SCID.beige mice after T cell reconstitution. T cell-reconstituted BALB/cnude mice showed titers of anti-rabbit IgG that were comparable to titers in BALB/c mice, whereas mouse anti-rabbit IgG was absent in SCID.beige mice because of the lack of functional B cells. (b) The content of CD49b+/CD3+/CD45+ NKT cells and TCRγδ+/CD3+/CD45+ cells was determined in the spleen of BALB/c mice to ensure that the CD3 positive T cell mix that was used for reconstitution contained viable NKT cells and γδT cells. Indeed, the mix contained an average amount of 1.27% γδT cells and 3.09% NKT cells. (a) Two-way ANOVA test with Bonferroni post hoc test (*p<0.05), mean (+SD), (a) n=5 (b) n = 3.

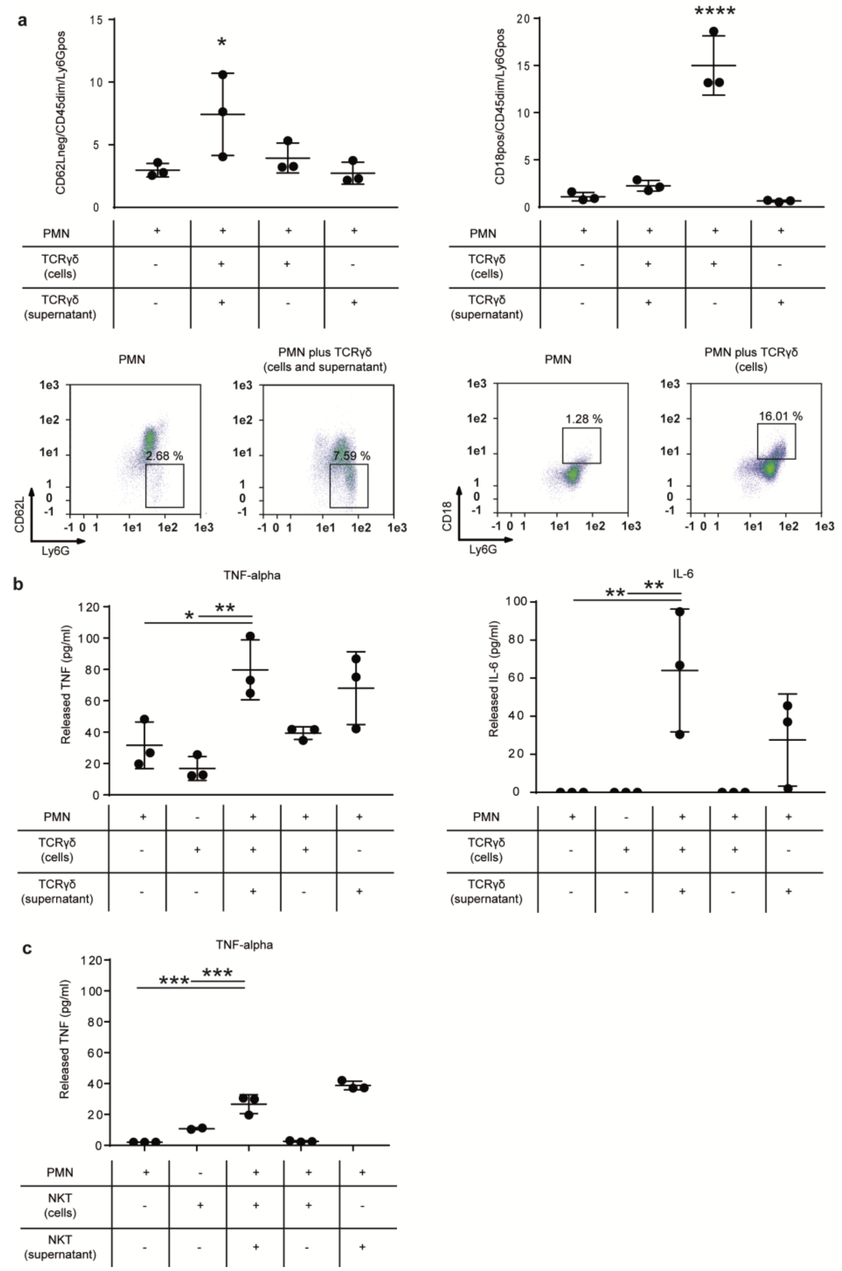

Supplementary Figure S5. TCRγδ or NKT cells directly affect neutrophil activation. TCRγδ or NKT cells were isolated from murine spleens and restimulated for 18 h. The cultured cells, the supernatant or the combination of both was subsequently co-cultured with freshly isolated murine neutrophils in a ratio of 1:4 for additional 4 h. The neutrophils were stained for FACS to evaluate the activation status. (a) Expression of CD18 is increased in cocultures of T cells and neutrophils. (b) The addition of TCRγδ induced CD62L shedding in neutrophils, whereas the supernatant of TCRγδ or the combination did not. (c) The supernatant of cultured cells was analyzed for cytokine release. The pro-inflammatory cytokines TNF and IL-6 were significantly higher in the co-culture then in cultured TCRγδ or neutrophils. (d) Isolated NKT cells that were co-cultured with neutrophils showed the same tendency for neutrophil stimulatory activities, but less pronounced. Only IL-6 could was significantly higher in the co-culture then in cultured NKT or neutrophils. Two-way ANOVA test with Bonferroni post hoc test (*p<0.05), mean (+SD), n = 3.
